# Thyroid Abnormalities in Survivors of Childhood Cancer

**DOI:** 10.4274/jcrpe.1326

**Published:** 2014-09-05

**Authors:** Ayla Akca Çağlar, Aynur Oğuz, Faruk Güçlü Pınarlı, Ceyda Karadeniz, Arzu Okur, Aysun Bideci, Ülker Koçak, Hüseyin Bora

**Affiliations:** 1 Gazi University, Faculty of Medicine, Pediatrics, Ankara, Turkey; 2 Gazi University, Faculty of Medicine, Pediatric Oncology, Ankara, Turkey; 3 Gazi University, Faculty of Medicine, Pediatric Endocrinology, Ankara, Turkey; 4 Gazi University, Faculty of Medicine, Pediatric Hematology, Ankara, Turkey; 5 Gazi University, Faculty of Medicine, Radiation Oncology, Ankara, Turkey

**Keywords:** Childhood cancer survivors, Chemotherapy, Radiotherapy, Late effects, Thyroid

## Abstract

**Ob­jec­ti­ve:** To investigate the late side effects of childhood cancer therapy on the thyroid gland and to determine the risk factors for development of thyroid disorder among childhood cancer survivors.

**Methods:** One hundred and twenty relapse-free survivors of childhood cancer (aged 6-30 years) were included in this study. The diagnoses of patients were lymphoma, leukemia, brain tumor, rhabdomyosarcoma and nasopharyngeal carcinoma (NPC). The patients were divided into two groups depending on the treatment: group 1-chemotherapy (ChT) only (n=52) and group 2-combination therapy of ChT + radiotherapy (RT) (head/neck/thorax) (n=68). Thyroid function tests, urinary iodine levels, and thyroid gland ultrasound examinations were evaluated in both groups.

**Results:** Incidence of thyroid disease was 66% (n=79) in the survivors. The thyroid abnormalities were: hypothyroidism (HT) (n=32, 27%), thyroid nodules (n=27, 22%), thyroid parenchymal heterogeneity (n=40, 33%), autoimmune thyroiditis (n=36, 30%), and thyroid malignancy (n=3, 2%). While the incidence of HT and thyroid nodules in group 2 was significantly higher than in group 1, the incidence of thyroid parenchymal heterogeneity and autoimmune thyroiditis was similar in the two patient groups. HT and thyroid malignancy were seen only in group 2. In multivariate logistic regression analysis, a history of Hodgkin lymphoma (HL), brain tumor and NPC, as well as cervical irradiation and 5000-5999 cGy doses of radiation were found to constitute risk factors for HT. History of HL and 4000-5999 cGy doses of radiation were risk factors for thyroid nodules. Head/neck irradiation and treatment with platinum derivatives were risk factors for autoimmune thyroiditis. In univariate analysis, a history of NPC, cervical + nasopharyngeal irradiation, and treatment with platinum derivatives were risk factors for thyroid parenchymal heterogeneity.

**Conclusion:** Our results indicate that there is especially an increased risk of HT and thyroid nodules in patients treated with combination therapy of ChT with head/neck/thorax RT. Although chemotherapeutic agents per se do not seem to cause HT, longer follow-up is needed to assess whether or not there is an increased risk for autoimmune thyroiditis and thyroid parenchymal heterogeneity after antineoplastic therapy.

## INTRODUCTION

Improvement in treatment of childhood malignancies has led to a decrease in mortality, and the overall 5-year survival rate for childhood cancers is now around 80% (1). This decrease has also led to a change in the scope of research, which at present focuses on detection of long-term side effects of treatment modalities.

Endocrine disorders are the most common long-term complication of cancer therapy. The side effects of this therapy on the endocrine system involve the hypothalamo-pituitary axis, the thyroid gland and the gonads (2). Thyroid dysfunction has been reported especially following head and neck irradiation (3,4,5). Although primary subclinical (compensated) hypothyroidism (HT) is the most common complication of this therapy, overt HT, Graves’ disease, benign nodules and thyroid malignancies can also occur after cervical radiotherapy (RT) (6,7,8). While the risk of thyroid dysfunction following RT has been described, the effect of chemotherapeutic agents on the thyroid gland has not yet been as well characterized.

In this study, our aim was to investigate the late side effects of childhood cancer therapy on the thyroid gland and to determine the risk factors for development of thyroid disorder among childhood cancer survivors.

## METHODS

The study included 120 relapse-free survivors of childhood cancer (84 male, 36 female, aged 6-30 years) who have been followed between November 1992 and October 2010 in the Department of Pediatric Hematology and Oncology at Gazi University Medical Faculty in Ankara, Turkey. The patients were divided into two groups depending on the type of treatment: Group 1-chemotherapy (ChT) only and Group 2-combination therapy of ChT with RT (head/neck/thorax). Group 1 consisted of 52 patients with diagnoses of non-Hodgkin lymphoma (NHL; n=27), leukemia (n=23) and HL (n=2). Group 2 consisted of 68 patients with diagnoses of HL (n=41), brain tumor (n=7), rhabdomyosarcoma (RMS; n=6), nasopharyngeal carcinoma (NPC; n=6), leukemia (n=5), NHL (n=2), retinoblastoma (n=1) and pleuropulmonary blastoma (n=1). Informed consent was obtained from all patients or parents. The research protocol was approved by the medical ethics committee.

Thyroid function studies including thyroid-stimulating hormone (TSH), plasma total thyroxine (tT4), free T4 (fT4), thyroglobulin (TG), thyroid antibodies (AB) [anti-thyroglobulin (anti-TG) and anti-thyroid peroxidase (anti-TPO)] and urinary iodine levels were measured in all patients. Normal values for these parameters were: TSH: 0.55-4.78 µU/L, tT4: 4.87-11.72 µg/dL, fT4: 0.74-1.52 µg/dL, Tg: 1.6-59.9 ng/mL, anti-TPO: 0-57 U/mL and anti-Tg: 0-64 U/mL. Plasma TSH, fT4, tT4 and AB levels were measured by chemiluminescence immunoassay (Siemens Immulite 2000). Tg was also determined by chemiluminescence immunoassay (Siemens-Centaur). Primary overt HT was defined as a low fT4 level and an elevated TSH. Compensated HT was defined as a high TSH level with a normal fT4 value. Central HT was diagnosed when accompanied by a low serum T4 with low or normal TSH levels. The presence of AB was accepted to indicate autoimmune thyroiditis. Urinary iodine levels were measured by spectrophotometry and levels ≥10 µg/dL were accepted as normal. Severe iodine deficiency was defined as urinary iodine levels lower than 2 µg/dL. Moderate iodine deficiency was defined as a urinary iodine level between 2 and 4.9 µg/dL and mild iodine deficiency as 5-9.9 µg/dL. Thyroid gland ultrasound was also performed (Hitachi EVB-7500 HV) for the diagnosis of thyroid nodules and thyroid parenchymal heterogeneity.

Eligibility criteria for inclusion in the current analysis were: diagnosis of the cancer at age ≤18 years, treatment with ChT only or both ChT and RT (head/neck/thorax) and a time interval of ≥6 months after completion of the therapy. Exclusion criteria for the analysis were history of thyroid disorder prior to the cancer therapy, severe iodine deficiency, treatment with RT only and RT treatment in field outside the thyroid area.

**Statistics**

Statistical analyses were performed using SPSS 15.0. Numerical data are expressed as median, minimum and maximum. All results are given as frequencies. The student’s t-test was used for comparison of quantitative data and chi-square for qualitative data. Univariate analyses of the patient and treatment factors possibly associated with thyroid disease were performed using the logistic regression to derive the odds ratios and their chi-square p-values. Covariates were included in a multivariate analysis if they turned out to have p<0.05 in the univariate assessments. The time elapsed after diagnosis of primary cancer and development of events was estimated using the method of Kaplan and Meir (9). All statistical tests were two-tailed and statistical significance was considered when the p-value was less than 0.05.

## RESULTS

Patient characteristics are shown in (Table 1). Mean age at diagnosis of malignancy was 8.5 years (1-17 years), mean current age was 16.4 years (6-30 years) and mean time elapsed after completion of the therapy was 7.8 years (0.75-18 years).

Overall, 79 (66%) survivors had developed thyroid disease. Thyroid abnormalities were HT (n=32, 27%), thyroid nodules (n=27, 22%), thyroid parenchymal heterogeneity (n=40, 33%), autoimmune thyroiditis (n=36, 30%) and thyroid malignancy (n=3, 2%) (Table 2). Severe iodine deficiency was not detected in any patient. Four patients had moderate and 24 patients had mild iodine deficiency.

Primary HT was seen in 31 (26%) patients (27 compensated and 4 overt HT), whereas central HT was diagnosed in only 1 case (0.8%) with brain tumor. HT was found only in Group 2 patients (p=0.000) (Table 2). The median time to development of HT was 5 years (0.5-12.5 years).

Sonographic abnormalities of the thyroid were found in 54 (45%) patients and 27 (22%) patients had thyroid nodules (solitary nodules in 14 patients and multiple-in 13 patients). Among the patients with thyroid nodules detected by ultrasonography, 3 had thyroid malignancy with a median duration of 11.5 years after the therapy. The median time to presence of thyroid nodules was 9 years (1-17 years) after cessation of the therapy. Thyroid nodules were more common in Group 2 (p=0.000) (Table 2).

Frequency rates of HT and thyroid nodules and significant variables associated with HT and thyroid nodules in univariate logistic regression analysis are shown in (Table 3). 

Vinca alkaloids and alkylating agents were the most commonly used drugs in the survivors (in 97% and 92%, respectively), followed by corticosteroids (88%), antineoplastic antibiotics (85%), antimetabolites (54%), intrathecal therapy (50%), asparaginase (27%), platinum derivatives (7%) and others (9%). Treatment with any type of chemotherapeutic agents did not increase the risk for developing HT and thyroid nodules. Also, patients with a history of NHL and leukemia, treatment with antineoplastic antibiotics, antimetabolites, asparaginase and intrathecal therapy were less prone to develop HT and thyroid nodules (Table 3).

Incidence of thyroid parenchymal heterogeneity and autoimmune thyroiditis was similar in Group 1 and Group 2 (Table 2). Frequency rates of thyroid parenchymal heterogeneity and autoimmune thyroiditis and significant variables associated with thyroid parenchymal heterogeneity and autoimmune thyroiditis in univariate logistic regression analysis are shown in (Table 4).

The results of multivariate logistic regression analysis of the significant variables associated with HT, thyroid nodules and autoimmune thyroiditis are shown in (Table 5). There was no significant variable associated with parenchymal heterogeneity in the multivariate logistic regression analysis.

## DISCUSSION

In our patient groups, 27% of the patients had HT, similar to the incidence reported by Madanat et al (10) and Van Santen et al (11). In line with several studies which have documented a higher incidence for subclinical HT as compared to overt HT (12,13,14), we also observed a higher rate of subclinical HT.

HT will usually occur within the first 5 years of treatment, with the peak occurring 2-3 years after the therapy. However, occurrences as late as 20 years and as early as within 3 months after the completion of therapy, were also reported (15,16). Our results showed a median interval of 5 years between completion of therapy and occurrence of HT.

Recent studies showed that female sex and younger age at diagnosis of malignancy are independent risk factors for HT in irradiated patients (4,10,13,15,17,18,19). However, we found no significant relationship between gender, age at diagnosis of malignancy and evidence of any thyroid disorder, except for autoimmune thyroiditis, which was more common in girls.

In patients with HL, the reported incidence of primary HT vary from 25% to 88% (4,12,15,19,20,21,22,23). In our study, the incidence of primary HT among the 43 HL patients was 51% (22 patients). Radiation therapy to the neck is a well-known risk factor for subsequent thyroid disease (12,13,15,18,24,25). A recent study by Bhatia et al (21) reported mantle irradiation with an estimated relative risk of 9.9 for the development of HT. In our series, we found that cervical, mantle and cervical + nasopharyngeal irradiation were all risk factors leading to HT.

The risk of HT is greatest after therapy for HL where RT 3500-4500 cGy is often used (13,15,25). In our study, we found an elevated risk for HT in patients with HL treated with doses of 2000-2999 cGy as well as in patients with brain tumor, NPC and RMS treated with ≥5000 cGy doses of radiation.

In children with brain tumor, primary HT is more prevalent than central HT. Paulino et al (17) documented primary HT in 38% and central HT in 19% of patients with brain tumor. Our study confirms previous reports indicating that prevalence of primary HT was greater than central HT in patients with brain tumor. This reflects the fact that the thyroid gland may receive some degree of radiation scatter in the course of cranial and spinal RT.

The effects of RT on the thyroid gland are well documented in survivors of childhood cancer. However, the side effects of ChT on thyroid function are still being debated. Chemotherapeutic agents were found to be associated with an increased risk of thyroid dysfunction in several studies (3,10,15,16,17,25,26,27,28,29), while other studies reported no such results (4,5,11,12,13, 14,20,21,22,24,30,31,32,33,34,35,36,37,38). In our study, chemotherapeutic agents were not found to increase the incidence of HT.

It has also been shown that RT involving the head and neck, particularly when applied during childhood, increases the risk of developing subsequent benign or malignant thyroid masses (4). Solt et al (36) reported an incidence of 54% of morphological abnormalities of thyroid gland detected by ultrasonography among HL patients who underwent mantle irradiation. Soberman et al (39) reported ultrasound-detected abnormalities in 89% of 18 long-term survivors of HL at a mean time interval of 6.4 years. In our series, thyroid nodules were seen in 27 of 120 survivors (22%) at a median interval of 9 years after the therapy and the frequency of ultrasound-detected thyroid nodules was significantly higher among patients treated with combination therapy than in those who had received ChT alone. We found a significant association of thyroid nodules with a history of HL and mantle irradiation. 

A dose-response relationship between radiation exposure to the thyroid and thyroid nodules was reported in one study in which survivors who received an estimated dose of 15-30 Gy to the thyroid gland were 6.0 times and those who received estimated dose of 31-66 Gy to the thyroid gland were 8.2 times more likely to develop nodules than patients receiving an estimated dose of <2 Gy to the thyroid gland (40). We also found that treatment with 4000-5999 cGy doses of radiation were associated with increased risk of developing thyroid nodules.

Some recent studies reported abnormalities detected by ultrasonography in the thyroid gland after RT (40,41) and several studies showed that RT can induce autoimmune thyroiditis (12,25). Tamura et al (25) reported that the incidence of AB positivity was less frequent in patients who had also received ChT than in patients who had received RT only, so they proposed that ChT, through its immunosuppressive effect, might control autoimmune thyroiditis induced by irradiation. In our study however, patients who were treated with combination therapy had a similar incidence of thyroid parenchymal heterogeneity and autoimmune thyroiditis as patients treated with ChT only. The incidence of autoimmune thyroiditis in two of our patient groups was found to be higher than that reported in Turkish children (42). Our study is the first to describe thyroid parenchymal heterogeneity and autoimmune thyroiditis in patients treated during childhood with ChT only. As autoimmune disorders tend to be transformed in lymphoproliferative diseases (43), this result may be due to either the disease itself or to the effect of ChT.

Thyroid parenchymal heterogeneity and autoimmune thyroiditis were significantly associated with treatment with platinum derivatives. Although seven of nine children treated with platinum derivatives also received RT, we suggest that the toxic effects of ChT may be independent of those of RT.

In conclusion, our results indicate an increased risk of HT and thyroid nodules in patients treated with combination therapy of ChT with head/neck/thorax RT. Chemotherapeutic agents do not seem to cause HT as an adverse late effect. In view of the similar frequency of autoimmune thyroiditis and thyroid parenchymal heterogeneity between patients treated with ChT only and combination therapy, longer follow-up is needed to assess whether or not there is an increased risk for these abnormalities after antineoplastic therapy.

## Figures and Tables

**Table 1:**
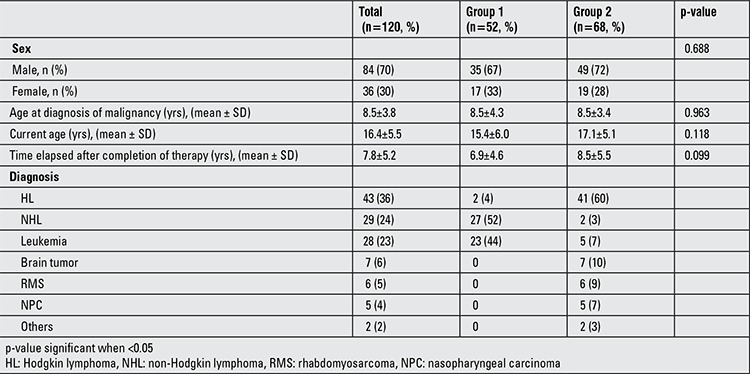
Patient characteristics according to diagnosis

**Table 2:**
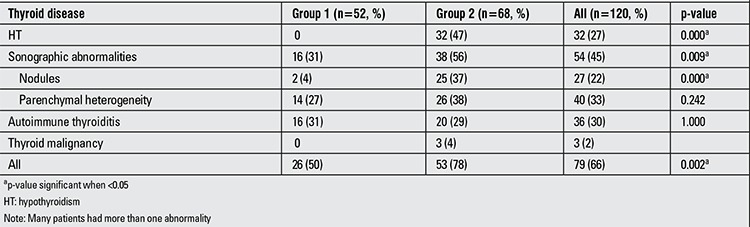
Occurrence of thyroid disease in each patient group

**Table 3:**
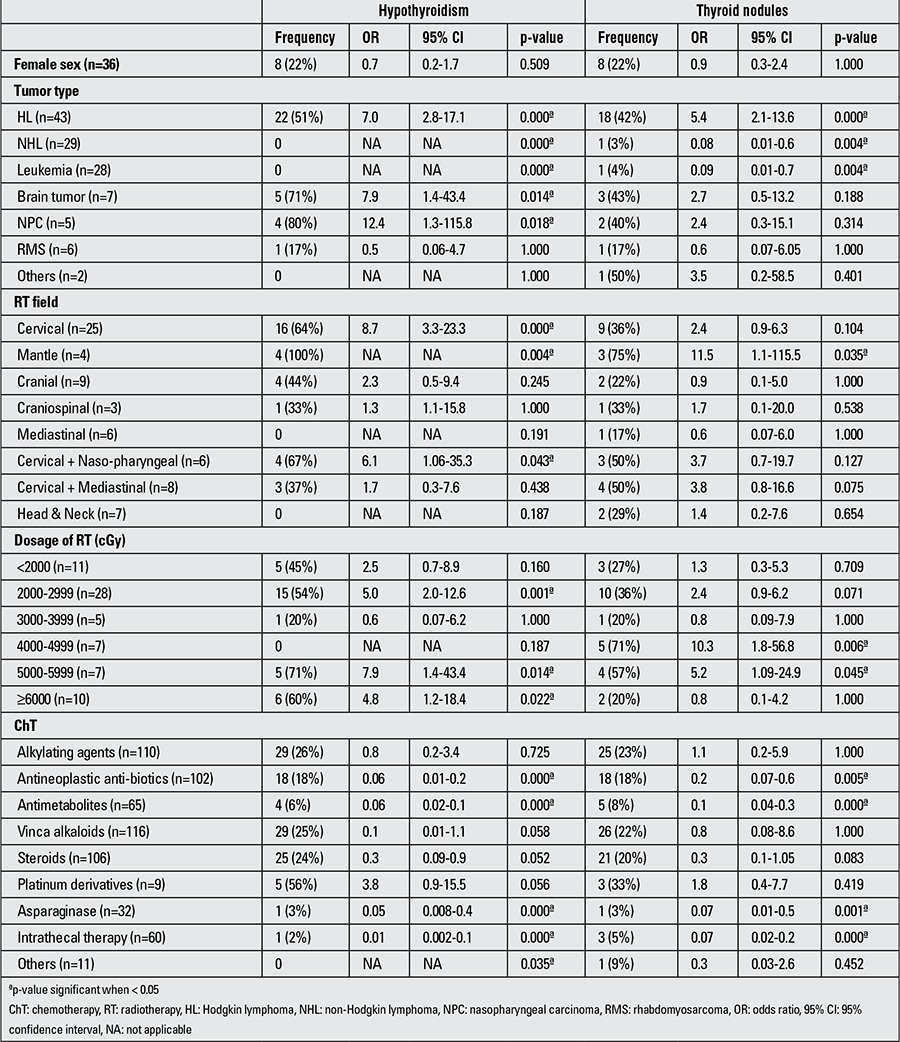
Univariate analysis of risk factors for developing hypothyroidism and thyroid nodules

**Table 4:**
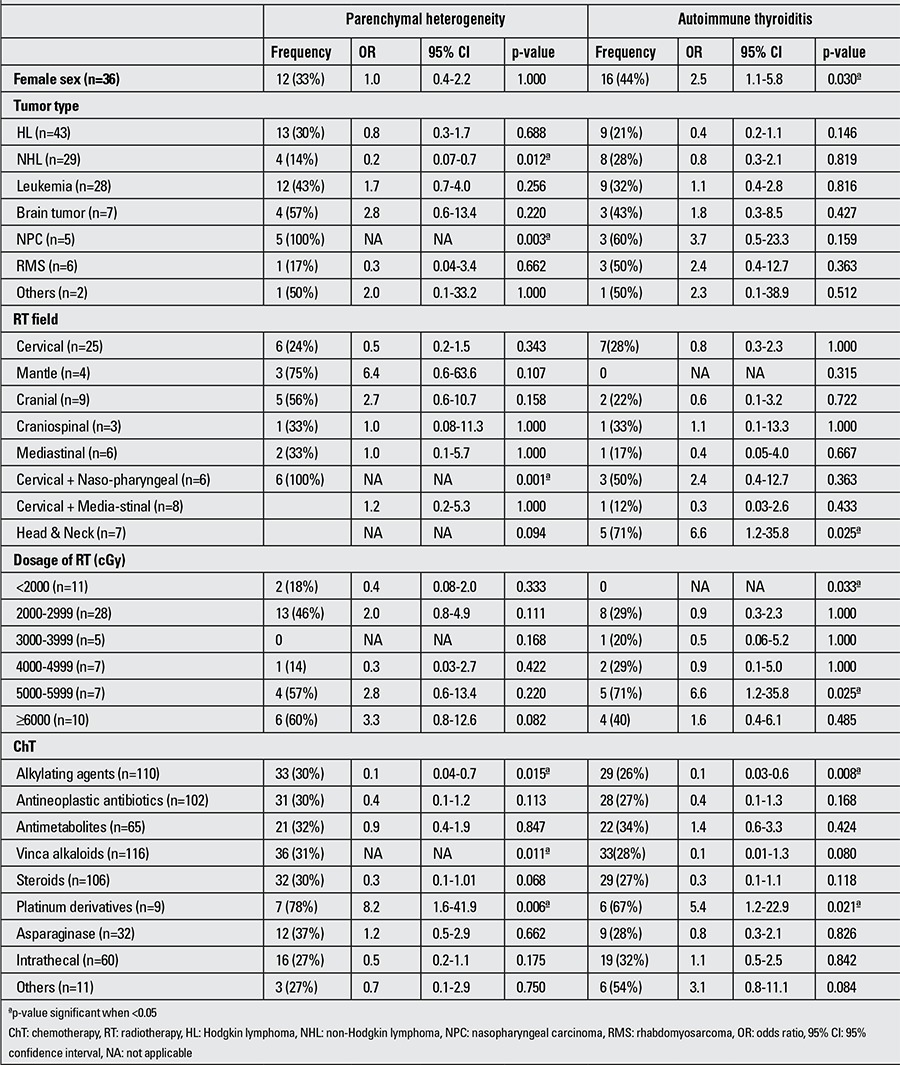
Univariate analysis of risk factors for developing parenchymal heterogeneity and autoimmune thyroiditis

**Table 5:**
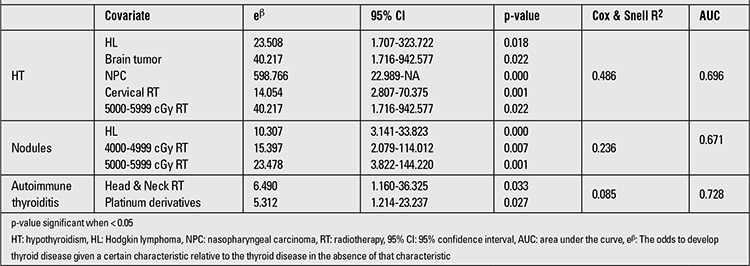
Multivariate logistic regression analysis of significant risk factors for developing thyroid disease
